# Optimization and validation of multi-coloured capillary electrophoresis for genotyping of *Plasmodium falciparum *merozoite surface proteins (*msp1 *and *2*)

**DOI:** 10.1186/1475-2875-8-78

**Published:** 2009-04-23

**Authors:** Anne Liljander, Lisa Wiklund, Nicole Falk, Margaret Kweku, Andreas Mårtensson, Ingrid Felger, Anna Färnert

**Affiliations:** 1 Department of Medicine Solna, Karolinska Institutet, SE-171 76 Stockholm, Sweden; 2 Swiss Tropical Institute, Socinstrasse 57, CH-4002 Basel, Switzerland; 3 Infectious and Tropical Diseases Department, London School of Hygiene and Tropical Medicine, London WC1E 7HT, UK; 4 Department of Public Health Sciences, Karolinska Institutet, SE-171 77 Stockholm, Sweden

## Abstract

**Background:**

Genotyping of *Plasmodium falciparum *based on PCR amplification of the polymorphic genes encoding the merozoite surface proteins 1 and 2 (*msp1 *and *msp2*) is well established in the field of malaria research to determine the number and types of concurrent clones in an infection. Genotyping is regarded essential in anti-malarial drug trials to define treatment outcome, by distinguishing recrudescent parasites from new infections. Because of the limitations in specificity and resolution of gel electrophoresis used for fragment analysis in most genotyping assays it became necessary to improve the methodology. An alternative technique for fragment analysis is capillary electrophoresis (CE) performed using automated DNA sequencers. Here, one of the most widely-used protocols for genotyping of *P. falciparum msp1 *and *msp2 *has been adapted to the CE technique. The protocol and optimization process as well as the potentials and limitations of the technique in molecular epidemiology studies and anti-malarial drug trials are reported.

**Methods:**

The original genotyping assay was adapted by fluorescent labeling of the *msp1 *and *msp2 *allelic type specific primers in the nested PCR and analysis of the final PCR products in a DNA sequencer. A substantial optimization of the fluorescent assay was performed. The CE method was validated using known mixtures of laboratory lines and field samples from Ghana and Tanzania, and compared to the original PCR assay with gel electrophoresis.

**Results:**

The CE-based method showed high precision and reproducibility in determining fragment size (< 1 bp). More genotypes were detected in mixtures of laboratory lines and blood samples from malaria infected children, compared to gel electrophoresis. The capacity to distinguish recrudescent parasites from new infections in an anti-malarial drug trial was similar by both methods, resulting in the same outcome classification, however with more precise determination by CE.

**Conclusion:**

The improved resolution and reproducibility of CE in fragment sizing allows for comparison of alleles between separate runs and determination of allele frequencies in a population. The more detailed characterization of individual *msp1 *and *msp2 *genotypes may contribute to improved assessments in anti-malarial drug trials and to a further understanding of the molecular epidemiology of these polymorphic *P. falciparum *antigens.

## Background

Genotyping of *Plasmodium falciparum *parasite populations is performed in order to determine the types and number of parasite clones in an infection. In molecular epidemiological studies of malaria, genotyping is used to study the genetic diversity of infections in relation to various factors such as transmission intensity and host immunity. The method enables studies of parasite population dynamics and tracking of individual clones over time. Genotyping is, therefore, recommended in anti-malarial drug trials to define treatment outcome by distinguishing recrudescent parasites from new infections [1].

The most widely used techniques for genotyping *P. falciparum *infections are based on polymerase chain reaction (PCR) amplification of the polymorphic genes encoding the merozoite surface proteins 1 and 2 (MSP1 and MSP2) and the glutamate-rich protein (GLURP) [2-8]. Since these are single copy genes, amplification generates DNA fragments corresponding to individual parasite clones. The methods used for genotyping *msp1 *and *msp2 *often include a two-step reaction with a primary amplification of the entire polymorphic gene segments, block 2 of *msp1 *and block 3 of *msp2*, followed by a nested reaction targeting the allelic type-specific regions within these blocks [4,5,7,8]. The PCR products are usually distinguished from each other based on fragment size after being separated by gel electrophoresis and visualization with ethidium bromide staining and UV transillumination. Interpretation of agarose gels and comparisons between separate runs is, however, not always straightforward, since differences between fragments and exact base pair (bp) size variations are often difficult to detect using the naked eye or even with digital software analysis. Indeed, variations in fragment migration and gel resolution stress the need for more specific and reproducible methods.

An alternative technique for fragment analysis is capillary electrophoresis (CE) performed using automated DNA sequencers. When applied to genotyping, the method is based on PCR amplification with fluorescently-labeled oligonucleotide primers, followed by fragment separation by electrophoresis in fine capillaries and detection by laser. The fragments' relative base-pair sizes are estimated in relation to the migration time of an internal fluorescent size standard using specific software. Allelic types are distinguished using primers labeled with different fluorescent dyes, which are detected as different colours upon laser excitation.

CE has successfully been used for typing several different microorganisms including *Mycobacterium tuberculosis*, *Legionella pneumophilia, Bacillus anthracis*, and *Escherichia coli *[9-12]. The technique has also been applied to the genotyping of *P. falciparum msp2 *polymorphism, both in an allele type-specific [13] and non-specific manner [14,15]. Compared to a PCR-based restriction fragment length polymorphism method (PCR-RFLP), a CE-based nested genotyping method revealed an improved resolution while detecting a higher number of *msp2 *genotypes per infection in a high transmission area [13]. Another CE-based assay, based on single *msp2 *amplification, has also been used for quantifying individual clones in multiclonal infections [14,15]. The potential of CE-based techniques has led to a recommendation by experts in the field to use automated sequencers for fragment analysis within the Medicines for Malaria Ventures/World Health Organization (MMV/WHO) protocol for genotyping in clinical trials on anti-malarial drug efficacy [1].

Here, one of the most widely used assays for genotyping *P. falciparum *populations, using nested allelic type specific amplification of both *msp1 *and *msp2 *[7,16] was adapted to CE. The CE-based method revealed several advantages compared to the standard gel electrophoresis for determining fragment size including improved resolution and allelic determination, and high throughput. Here the procedure and the optimization process as well as the potentials and limitations of the technique in molecular epidemiology studies and anti-malarial drug trials are reported.

## Methods

### Laboratory cultured parasite lines

The laboratory-cultured *P. falciparum *lines F32, K1, 7G8 and TM180 were used as positive controls. Parasite DNA was extracted from red blood cell cultures using E.N.Z.A Blood DNA Kit (Omega Bio-Tek, Inc. Doraville, GA, USA) according to the manufacturer's instructions.

### Field samples

#### Intermittent preventive treatment trial

Finger prick blood samples collected on filter paper from 240 microscopy-positive children between three and 59 months of age, during a trial of intermittent preventive treatment (IPTc) in Hohoe, Ghana [17] were genotyped in order to evaluate the method in natural infections in a highly endemic area. DNA was extracted from whole blood spots using ABI Prism 6100 Nucleic Acid PrepStation (Applied Biosystems, Foster City, CA, USA). Written consents were obtained from the participating children's caregivers. Ethical approval was received from the Ethical Review Committee of Ghana Health Services and from the Regional Ethical Review Board in Stockholm, Sweden.

#### Anti-malarial drug trial

Finger prick blood samples collected on filter paper from 57 children, below 5 years of age, with acute, uncomplicated *P. falciparum *malaria participating in an efficacy trial of artesunate + amodiaquine versus artemether-lumefantrine in Zanzibar, Tanzania (Mårtensson et al, *manuscript in preparation*), were genotyped in order to evaluate the method in an anti-malarial drug trial. Paired blood samples collected before the initiation of treatment and at recurrent parasitaemia between days 21–42 of follow up were analysed (n = 114). DNA was extracted from blood spots using ABI Prism 6100 Nucleic Acid PrepStation (Applied Biosystems). Informed consent was obtained from parents of enrolled children. The study obtained ethical approval from the Zanzibar Medical Research Council and from the Regional Ethical Review Board in Stockholm, Sweden.

### Original *msp1 *and *msp2 *genotyping assays

#### PCR method (non-fluorescent)

Genotyping of *P. falciparum *parasites was performed with a nested PCR assay based on the amplification of *msp1 *and *msp2 *as described in detail elsewhere [7,16] with some modifications. In brief, in the primary reaction, the oligonucleotide primers span the entire genetic segments, block 2 for *msp1 *and block 3 for *msp2 *(Table 1). In the nested reaction, separate primer pairs target the respective allelic types of *msp1 *(K1, MAD20, and RO33) and *msp2 *(FC27 and IC – elsewhere also referred to as 3D7) (Table 1).

**Table 1:** Oligonucleotide primers for *msp1 *and *msp2 *genotyping and fluorescent modifications for the CE method

Primer sequences^a^		Fluorophore modification^b^
Segment-specific primers for block 2 of *msp1 *and block 3 of *msp2*		
		
In the primary reaction		
*msp1 *F	5'-CTAGAAGCTTTAGAAGATGCAGTATTG-3'	
*msp1 *R	5'-CTTAAATAGTATTCTAATTCAAGTGGATCA -3'	
*msp2 *F	5'-ATGAAGGTAATTAAAACATT GTCTATTATA-3'	
*msp2 *R	5'- CTTTGTTACCATCGGTACATTCTT-3'	
		
Allelic type-specific primers for *msp1 *in the nested reaction		
K1 F	5'- AAATGAAGAAGAAATTACTACAAAAGGTGC-3'	7 bp-tail
K1 R	5'- GCTTGCATCAGCTGGAGGGCTTGCACCAGA-3'	NED™ (yellow)
MAD 20 F	5'- AAATGAAGGAACAAGTGGAACAGCTGTTAC -3'	7 bp-tail
MAD 20 R	5'- ATCTGAAGGATTTGTACGTCTTGAATTACC'-3'	PET^® ^(red)
RO33 F	5'- TAAAGGATGGAGCAAATACTCAAGTTGTTG-3'	7 bp-tail
RO33 R	5'- CATCTGAAGGATTTGCAGCACCTGGAGATC-3'	VIC^® ^(green)
		
Allelic type-specific primers for *msp2 *in the nested reaction		
FC27 F	5'- AATACTAAGAGTGTAGGTGCARATGCTCCA-3'	7 bp-tail
FC27 R	5'- TTTTAT TTG GTGCAT TGCCAGAAC TTG AAC-3'	6-FAM™ (blue)
IC ^c ^F	5'- AGAAGTATGGCAGAAAGTAAKCCTYCTACT-3'	7 bp-tail
IC^c ^R	5'- GATTGTAATTCGGGGGATTCAGTTTGTTCG-3'	VIC^® ^(green)

^a ^Primer sequences according to the original method [7,16]

^b ^Modification of the primers used in the CE-based method

^c ^Also referred to as 3D7 type

F = forward R = reverse

#### Primary reaction

The final concentration of the master mix consisted of 1× PCR buffer, 2 mM MgCl_2_, 125 μM dNTP and 0.02 units/μl of AmpliTaq^® ^DNA polymerase (Applied Biosystems), and 250 nM each of the outer primer pairs *msp1 *forward (F)/reverse (R) and *msp2 *F/R. For the laboratory lines, 2 μl DNA was used as a template with the volume corresponding to 1 μl of whole blood. The amount of field sample DNA used was 2 μl and 3 μl from Zanzibar and Ghana, respectively corresponding to 0.1–0.3 μl whole blood assuming a blood volume per spot of 10–20 μl. The cycle conditions were: initial denaturation at 95°C for 5 min followed by 25 cycles of annealing at 58°C for 2 min, extension at 72°C for 2 min, denaturation at 94°C for 1 min with a final round of 58°C for 2 min and 72°C for 5 min.

#### Nested reaction

The final concentration of the master mix consisted of 1× PCR buffer, 1 mM MgCl_2_, 125 μM dNTP and 0.02 units/μl of AmpliTaq^® ^DNA polymerase, and 250 nM of the respective *msp1 *allelic type-specific primers (K1, MAD20, and RO33 types) and 125 nM of the respective *msp2 *type primers (FC27 and IC types) in separate reactions.

For the respective nested reactions, 1 μl product from each primary reaction was used as a template. For the amplification of the *msp1 *allelic types, the cycle conditions were as follows: initial denaturation at 95°C for 5 min followed by 30 cycles of annealing at 61°C for 2 min, extension at 72°C for 2 min, denaturation at 94°C for 1 min, and a final round at 61°C for 2 min and 72°C for 5 min. For the *msp2 *allelic types, the cycle conditions were as follows: initial denaturation at 95°C for 5 min, followed by 30 cycles of annealing at 58°C for 1 min, extension at 72°C for 1 min followed by 94°C for 30 sec, and a final round at 58°C for 1 min and 5 min at 72°C. All amplifications were performed on 96-well plates with a total reaction volume of 20 μl per well.

#### Fragment analysis by gel electrophoresis

The amplified products from the nested reaction were separated using electrophoresis on a 2% high resolution agarose gel (Agarose 3:1 HRB™, Amresco, Inc, Cleveland, OH, USA) in 1× TBE buffer (100 mM Tris, 100 mM boric acid, 5 mM EDTA [pH 8.0]) and, following staining with ethidium bromide, visualized with UV light in a Universal hood II (Bio-Rad Laboratories, Inc, Hercules, CA, USA). Fragment size was estimated in relation to a 100 base-pair DNA ladder (Invitrogen Corporation, Carlsbad, CA, USA) both by the naked eye and in a GelDoc Xr system with Quality One^® ^analysis software version 4.4.1 (BioRad).

### Modified genotyping assays for capillary electrophoresis

#### Fluorescent PCR method

The PCR protocol for the CE method was based on the *msp1 *and *msp2 *genotyping assay described above [7,16]. The primary PCR reaction was identical to the original assay. In the nested reaction, the allelic type-specific primers were modified as follows: (i) the forward primers were tailed with a 7-bp tail (Applied Biosystems) at the 5'-end in order to promote the non-template adenosine (A) addition by the *Taq *DNA polymerase at the 3' end of the PCR products [18], (ii) the reverse primers were labeled with different fluorophores at the 5'-end: *msp1 *K1 with NED™ (yellow), MAD 20 with PET^® ^(red), and RO33 with VIC^® ^(green); *msp2 *FC27 with 6-FAM™ (blue) and IC with VIC^® ^(green) (Table 1). The addition of the tail promoting the additional A counteracts amplification of fragments differing with a single nucleotide i.e. fragments ± A. Moreover, due to the addition of the tail on the forward primers, all GeneMapper^®^-estimated bp sizes presented in this paper include an extra 8 bp segment (7 bp tail +A).

#### Nested reaction

For the fluorescent assay, different modifications of the original nested reaction were evaluated. The final protocol included the following adjustments: (i) the concentration of all *msp1 *and the *msp2 *FC27 allelic type-specific primer pairs was decreased to 125 nM each (F/R); (ii) in the *msp2 *IC reaction, the primers were increased to 300 nM each (F/R) and the AmpliTaq^® ^DNA polymerase to 0.05 units/μl; (iii) the number of cycles was reduced to 23 in all nested fluorescent PCR amplifications. All other concentrations and conditions were kept identical to the original nested reaction.

#### Fragment analysis by capillary electrophoresis

Fragment analysis was performed on a 3730 DNA sequencer (Applied Biosystems) equipped with 48 capillaries (36 cm), using POP-7™ polymer (Applied Biosystems). From the nested reaction, 1 μl product was added to 9 μl Hi-Di formamide (Applied Biosystems) and 0.5 μl size standard (GS™-LIZ^® ^1200, Applied Biosystems) per well on 96-well plates. Resulting peaks were diluted 1:10 or 1:20 in sterile water after initial screening to achieve peaks < 8000 rfu. The size standard contains 73 single-stranded DNA fragments ranging in size from 20 bp to 1200 bp.

The *msp1 *and *msp2 *markers were run separately during CE, due to competition for separation between the smaller VIC-labeled *msp1 *RO33 fragments and the larger VIC-labeled *msp2 *IC fragments. The sample volume in the capillary system was adjusted depending on the number of allelic types analysed simultaneously; 10.5 μl when the respective allelic types were run separately, 11.5 μl for multiplex *msp2 *(FC27 + IC), and 12.5 μl for multiplex *msp1 *(K1+MAD20+RO33). The separation was run at 8.0 kV for 4000 sec. The results were interpreted using GeneMapper^® ^Software version 4.0 (Applied Biosystems).

#### Multiplex versus simplex amplification in the fluorescent nested reaction

To increase the CE method's throughput, a multiplex approach to the nested PCR reaction was evaluated. The allelic types were run separately for *msp1 *and *msp2 *and in mixtures (multiplex *msp1 *and *msp2*). The allelic type specific primers were mixed accordingly; *msp1 *K1+MAD20+RO33, *msp2 *FC27+IC. The risk of hybrid and artifact products was also evaluated in an *msp2 *type-specific hybrid assay (FC27 F-7 bp tail/IC R-VIC and FC27 R-6-FAM/IC F-7 bp tail) in two separate nested reactions. The amount of AmpliTaq^® ^DNA polymerase was increased to 0.05 units/μl in the multiplexed and in the hybrid assays. All other concentrations and conditions were kept identical to the fluorescent nested reaction. DNA suspensions from laboratory lines (F32, K1, and 7G8) corresponding to 1000, 50 and 10 parasites/μl, respectively, and a set of field samples from Ghana (n = 81) were analysed. The multiplex and simplex nested reactions were both followed by multiplexed CE for *msp1 *and *msp2*. All PCR products were diluted 1:10 before carrying out CE.

### Determination of sensitivity, specificity, and reproducibility

DNA from laboratory lines (F32, K1, and 7G8) were diluted stepwise in sterile water, to concentrations corresponding to 5,000, 1,000, 500, 100, 50, 10, 5 and 1 parasites/μl whole blood (assuming a haematocrit of 50%), and mixed in different proportions (10:1,000, 500:500, and 1,000:10 parasites/μl). All PCR amplifications were repeated at least twice to assess the reproducibility of detection and size determination.

The *msp1 *and *msp2 *nested amplicons from the laboratory lines were sequenced to validate the accuracy of size calling of CE. The PCR products were purified using Montage^® ^PCR Filter Units (Millipore, Billerica, MA, USA) prior to sequencing (performed by Uppsala Genome Centre, Uppsala, Sweden). Since fluorescently-labeled PCR products cannot be sequenced due to dye interference, the original allelic type-specific primers were used for amplification before sequencing. The sequences were analysed using the DNA Sequencing Analysis Software version 5.2 (Applied Biosystems).

The accuracy of relative quantification by CE-based genotyping was evaluated by calculating the ratios between the area under the curve (AUC) values, corresponding to the amount PCR product, in mixtures of laboratory lines (99:1, 50:50, 1:99) of the same or different *msp2 *allelic types, thus labeled with the same or different fluorescent dyes. The ratio between alleles was expressed as AUC value for one allele/total AUC values for all alleles within that sample. The calculated proportions were compared with the actual proportions of the respective lines. All PCR products were diluted 1:20 before CE.

### Enumeration of clones in natural infections

The CE method was compared to gel electrophoresis for its ability to distinguish separate alleles and determine the number of concurrent clones in samples collected from 240 microscopy- positive children during an IPTc trial in Ghana. All fluorescent products were diluted 1:10 before CE. Fragments were allocated to size bins of 3 bp to determine allele frequencies.

### Distinguishing recrudescent from new infections

Paired samples (n = 114) from 57 children participating in an anti-malarial drug trial in Zanzibar, Tanzania, were genotyped to compare CE and gel electrophoresis in their ability to determine recrudescent versus new infections during follow-up after treatment. Genotyping was done stepwise with an initial *msp2 *followed by *msp1 *genotyping of the paired blood samples found to have at least one identical *msp2 *allele before and after treatment. Paired samples were placed randomly on the 96-well plates for analysis by CE, while being run on adjacent lanes on agarose gel. Using CE, alleles were considered to be the same if the fragments differed less than 1 bp. With gel electrophoresis, the fragments were compared using the naked eye and estimated to be of the same length.

## Results

### Fluorescent artifacts

The PCR assay with fluorescently labeled primers for the CE method was initially set up with the same conditions as the original gel electrophoresis method. Optimization of several parameters was, however, required. During the process different types of artifacts were identified on the electropherograms not detected on corresponding gels.

#### Fluorescent background and non-specific low background artifacts

**Figure 1:**
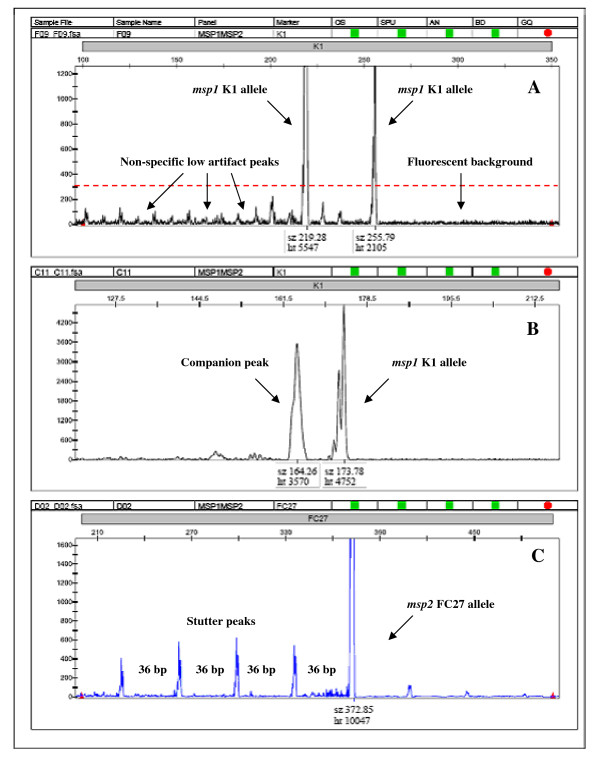
**Screenshot of electropherogram (GeneMapper^® ^software) displaying different artifacts: A) fluorescent background (right) and non-specific low background (left) in a field sample with 2 *msp1 *K1 alleles**. The red striped line indicates the cut-off at 300 relative fluorescent units (rfu). B) Companion peak, to the left of a true peak of an *msp1 *K1 allele from a single laboratory line. C) Stutter peaks in a sample with the K1 laboratory line amplified with *msp2 *FC27 primers. The y-axis depicts the rfu while fragment size (sz) in base pair and rfu height (ht) for individual alleles is depicted on the x-axis. The rfu scale on the y-axis has been adjusted to focus on the artifacts.

An inherent fluorescent background was detected in all CE runs (Figure 1A, right). The relative fluorescent unit (rfu) value for the background was generally below 50 units. A slightly higher background of non-specific artifact peaks was occasionally seen in field samples, independent of parasite densities (Figure 1A, left). This background was reduced by optimizing primer concentrations and the amount of DNA template. A cut-off value of 300 rfu [13] was introduced to simplify interpretation and identification of true alleles in the optimized assay.

#### Companion peaks

Artifact peaks with a typical characteristic were distinguished in samples known to contain single clones. These peaks were wider and rounder than the true allele peak, and were positioned 5 to 20 bp left of a true allele peak (Figure 1B). The rfu values of these peaks, which we termed "companion peaks", were often similar to, and even higher than, the true alleles. Occasionally, they appeared to reduce the rfu value of the true allele to values below cut-off. These artifacts were run-specific, that is, when present within a run they were present in most samples on that plate. However, these artifacts have only been seen in four CE runs so far. When the PCR products from the same samples were separated by gel electrophoresis, only the fragments corresponding to the true allele were detected, suggesting companion peaks to be purely fluorescent artifacts. Diluting the nested PCR products in water before CE removed the companion peaks and resulted in an increased rfu of the true alleles.

#### Stutter peaks

Amplification of laboratory lines at high parasite densities generated artifact peaks in strict repetitive patterns, stutter peaks, at base-pair intervals specific for the respective clones and allelic types. Amplification of *msp2 *FC27 of the K1 laboratory line generated peaks situated approximately 36 bp apart (Figure 1C). For the F32 clone, the *msp2 *IC amplification resulted in stutter peaks at 12 bp intervals, and for *msp1 *MAD20, 18 bp apart. This type of stutters was not detected in any of the *msp1 *K1 or RO33 amplifications. The stutter peaks were all of similar height and lower than the true allele (Figure 1C). In 18 *msp2 *runs of the K1 line in different dilutions, the FC27 type-specific stutter peaks never exceeded 6% of the rfu value of the true allele peak. In the only field sample in which a 36 bp pattern was detected, the rfu values of these FC27 type-specific stutter peaks were below 14% of the rfu of the true allele.

Another stutter pattern was seen in 17 of 54 samples from Ghana with a *msp2 *FC27-type peak of 383 bp accompanied by two smaller peaks at 96–97 bp intervals, at 286 bp and 189 bp. These peaks were never detected alone, and were all < 10% of the 383 bp allele height, except in one sample where they corresponded to 40% of the height. When these products were run on a gel, only one band corresponding to the 383 bp allele was seen, suggesting that the two smaller peaks were artifacts. In two samples from Ghana, an *msp1 *MAD20 allele, which was also detected as a single band on the gel, appeared with stutter peaks situated approximately 27 bp and 9 bp apart, respectively.

### Optimization of the fluorescent PCR assay and capillary electrophoresis

Several parameters in the fluorescent nested reaction and product preparation prior to CE were optimized in order to reduce the artifacts described above and ensure sustained sensitivity.

#### Primer concentration

Reduced primer concentrations (200, 150, 125, 100, and 75 nM) of the allelic type-specific primers in the nested reaction resulted in a considerable reduction in the height of true allele peaks as well as the stutters and non-specific low artifact peaks. Concentrations below 125 nM, however, affected detection sensitivity in low parasite density controls (5 parasites/μl). The primer concentration for all *msp1 *allelic types and for the *msp2 *FC27 was therefore set to 125 nM of F/R primers each.

The *msp2 *IC assay needed a more extensive optimization in order to attain satisfactory sensitivity. Different concentrations of MgCl_2 _and dNTP as well as annealing temperatures (55, 53 and 50°C) in the nested reaction did not affect the outcome. The optimal amplification with *msp2 *IC primers was achieved with a primer concentration of 300 nM of F/R each and 0.05 units AmpliTaq^® ^DNA polymerase.

To reduce the stutter peaks in the *msp2 *FC27 runs, high performance liquid chromatography (HPLC) purified (by manufacturer, Applied Biosystems) reverse primers (6-FAM-labeled) as well as AmpliTaq^®^Gold DNA Polymerase (Applied Biosystems) with hot-start activation were tested. Both approaches reduced the general peak height but did not considerably affect the presence of stutter peaks.

#### Amount of DNA template

Different amounts of DNA template (3 μl, 2 μl, and 1 μl) from the K1 line and field samples from Ghana (n = 32) were added in the primary PCR reaction. One μl of DNA generally resulted in fewer stutter peaks and non-specific low artifact peaks, however, also resulting in allele dropout and negative results in 8/35 otherwise positive samples. Although the amount of DNA affected the detection and number of peaks, it did not affect, to any considerable extent, the rfu height of peaks or the occurrence of stutters.

#### Number of PCR cycles

The relative fluorescent intensity of the stutter peaks decreased when the number of cycles in the nested reaction was reduced from 30 to 25 cycles. A further reduction to 20 cycles almost completely eliminated the artifacts, yet with a 10-fold reduction in sensitivity. A nested reaction with 23 cycles, for all *msp1 *and *msp2 *allelic types, was optimal with regards to sustained sensitivity and reduced artifacts.

#### Dilution of nested PCR products prior to capillary electrophoresis

According to the manufacturer (Applied Biosystems), an optimal analysis with the current genotyping assay should not result in peaks higher than 8000 rfu. Peaks exceeding 20 000 rfu, however, occurred in samples with a wide range of parasitaemias (range 120 to 98 600 parasites/μl). Dilution of the final products from the nested reaction in sterile water (1:5, 1:10, and 1:20) before carrying out CE resulted in reduced rfu values, as well as elimination of companion artifact peaks. Still, samples with low parasite concentrations (5 parasites/μl) were occasionally left undetected in 1:20 dilution, thus 1:10 dilutions were most commonly used.

### Increasing PCR throughput – multiplex versus simplex nested PCR

The ability to multiplex the nested reactions, that is, simultaneous amplification with all the respective allelic type-specific primers for *msp1 *and *msp2*, was evaluated on laboratory lines (F32, K1, and 7G8) and 81 field samples from Ghana. The same detection levels (10 and 50 parasites/μl) and the number of positive samples (n = 80) were obtained for the two markers by both, multiplex and simplex approaches, respectively. The number of detected *msp1 *fragments was similar by simplex (213 fragments) and multiplex PCR (215 fragments). For *msp2*, however, the multiplexed assay detected more FC27 fragments (101 vs. 79) and fewer IC fragments (136 vs. 149) compared to the simplex assay. The allelic type-specific primers were also combined (FC27 F-7 bp tail/IC R-VIC and FC27 R-6-FAM/IC F-7 bp tail) in separate reactions. In this hybrid assay, 16 additional fragments observed in multiplex PCR as well as 26 additional FC27 and 13 IC fragments were detected. Notably, the K1 clone, known to be a FC27 type and not representing a crossing over between allelic types, gave a positive result in the hybrid assay.

### Detection sensitivity and specificity

To compare the sensitivity of detection, serial dilutions and mixtures of the laboratory lines F32, K1, and 7G8 were analysed by CE and gel electrophoresis in the optimized assay. A detection sensitivity of 5–10 parasites/μl was found for both *msp1 *and *msp2 *markers by the respective methods (Table 2). In the mixtures, the dominating genotype, for example, 1000 parasites/μl was always detected by both methods. The low concentration genotype (10 parasites/μl), which was always detected in single clone samples, was more often detected by CE when mixed with another clone at a higher concentration (Table 2). The rfu values of the allele peaks were generally lower in runs with multiple compared to single clones.

**Table 2:** Genotyping of *msp1 *and *msp2 *in laboratory lines in different dilutions and mixtures using CE and gel electrophoresis

		*msp1*						*msp2*			
		K1		MAD20		RO33		FC27		IC	
Lines	Density^a^	CE^b^	gel^c^	CE^b^	gel^c^	CE^b^	gel^c^	CE^b^	gel^c^	CE^b^	gel^c^

F32	1000	0	0	192.99	205.50	0	0	0	0	524.09	539.10
	100	0	0	193.01	207.06	0	0	0	0	524.05	539.12
	50	0	0	192.94	203.59	0	0	0	0	524.07	539.28
	10	0	0	193.02	201.20	0	0	0	0	524.25	519.80
	5	0	0	192.99	205.10	0	0	0	0	524.06	540.91
	1	0	0	0	0	0	0	0	0	0	0
K1	1000	173.48	177.61	0	0	0	0	372.82	392.41	0	0
	100	173.43	180.28	0	0	0	0	372.85	378.61	0	0
	50	173.30	179.85	0	0	0	0	372.87	381.90	0	0
	10	173.64	182.89	0	0	0	0	372.86	382.01	0	0
	5	173.56	182.51	0	0	0	0	372.85	395.98	0	0
	1	0	0	0	0	0	0	0	0	0	0
7G8	1000	0	0	0	0	157.06	165.55	0	0	493.14	488.25
	100	0	0	0	0	157.35	162.95	0	0	493.13	488.21
	50	0	0	0	0	157.23	167.94	0	0	493.16	472.87
	10	0	0	0	0	157.33	160.40	0	0	493.19	515.93
	5	0	0	0	0	157.19	165.92	0	0	0	508.05
	1	0	0	0	0	0	0	0	0	0	0
F32/K1	1000/10	173.60	177.06	192.98	197.04	0	0	0	0	523.52	509.46
	500/500	173.66	174.49	192.96	194.87	0	0	372.99	380.85	524.25	521.31
	10/1000	173.69	182.86	193.02	192.74	0	0	372.96	379.48	524.19	519.79
K1/7G8	1000/10	173.64	178.01	0	0	157.31	152.59	372.97	364.85	493.51	483.32
	500/500	173.60	175.39	0	0	157.34	161.56	372.98	363.65	493.43	485.01
	10/1000	173.61	172.85	0	0	157.26	161.57	0	0	493.38	500.08
F32/7G8	1000/10	0	0	193.08	190.99	157.49	0	0	0	524.20/492.68	521.99/0
	500/500	0	0	193.08	188.05	157.49	165.59	0	0	524.19/493.47	539.46/0
	10/1000	0	0	193.07	183.33	157.52	163.02	0	0	524.08/493.41	0/493.20

^a ^parasites/μl

^b ^Fragment siz (bp) including 7 bp tail + A, determined by GeneMapper^® ^soft ware after simplex PCR

^c ^Fragment sizes (bp) determined by Quality One 4.4.1 analysis software

**Figure 2:**
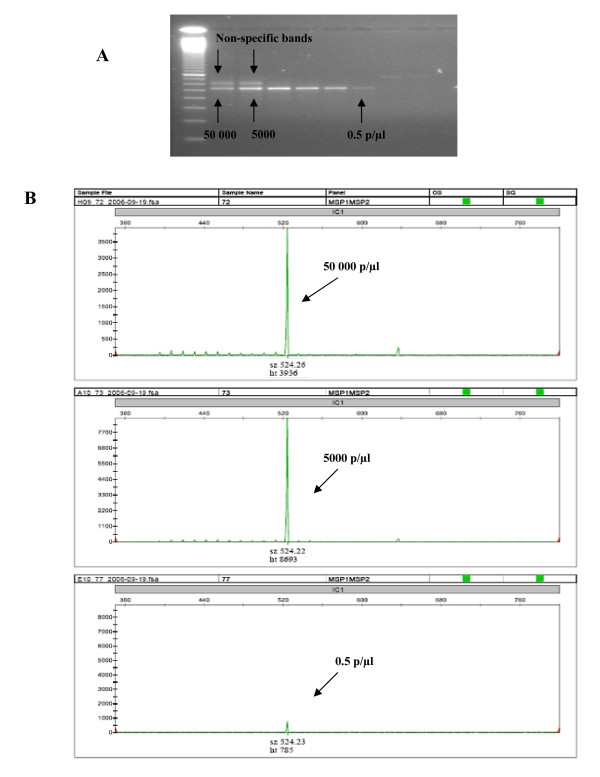
**Genotyping of *msp2 *of the F32 laboratory line in different concentrations exemplifies non-specific bands that often appear in high density samples following electrophoresis on agarose gel (A) and the corresponding electropherogram with a single peak corresponding to the F32 allele (B)**.

In high density samples (> 5000 parasites/μl), the gel method often generated non-specific bands and smears, whereas the CE-based method generated one single peak on the electropherograms irrespective of parasite density (Figure 2).

AUC-based quantification of proportions of laboratory lines of the same allelic types was most accurate in mixtures with two clones at 50:50 and at 99:1 ratios (Additional file 1, Table S1) however, mixtures with three clones at equal proportions resulted in highly inaccurate quantifications. An even higher disagreement between actual and observed compositions was found in mixtures of clones of different allelic types, that is, different dye labeling (Additional file 2, Table S2).

### Fragment sizing and reproducibility

**Figure 3:**
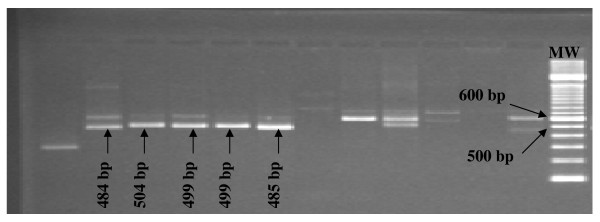
***Msp2 *IC-type fragments separated with electrophoresis on agarose gel and the corresponding bp size determined by CE and GeneMapper^® ^software**. MW: 100 base-pair DNA ladder.

The CE assay was able to precisely determine fragment sizes at the single base-pair level. This is illustrated in Figure 3 with five different *msp2 *IC type amplicons estimated by the naked eye to be approximately 500 bp on an agarose gel, and the corresponding precise base-pair lengths determined by GeneMapper^® ^following CE.

Determining the fragment size of the laboratory lines in repeated PCR and CE runs resulted in size variation < 0.5–1 bp (Table 2), demonstrating high reproducibility and precision. When the corresponding non-fluorescent products were analysed by software (Quality One), following separation on gel, the fragments of the same products varied by 2–16 bp when run in different lanes on the same gel or between different runs.

Sequencing was performed for the *msp1 *and *msp2 *products (non-fluorescent) from the F32, K1, 7G8 and TM180 lines. The fragment sizes determined by GeneMapper^® ^software differed by ≤ 4 bp from the determined sequences (including adjustments for the 7 bp tail and the additional A), indicating a high accuracy in sizing by the CE method.

The reproducibility of fragment sizing on different instruments was tested by running a CE analysis of the same fluorescent *msp2 *FC27 and IC products from 83 samples (77 samples from the Ghana field trial and 6 positive controls) on the 3730 DNA Analyzer available at the department and on a similar instrument at the Uppsala Genome Centre, Uppsala, Sweden. This resulted in similar precision in size determination of individual alleles (< 0.5 bp) between the instruments.

### Enumeration of clones in natural infections

The number of clones detected with the *msp1 *and *msp2 *markers using CE and gel electrophoresis was analysed in 240 microscopy-positive samples (parasite range 40–162240 parasites/μl) from children in an IPT trial in Ghana. Of these, 223 (93%) were positive for both markers using both methods. In 20 samples, stutter patterns were detected by CE, as described above: one sample with 36 bp FC27 stutter pattern, two samples with MAD20 stutter peaks at 27 bp and 9 bp, and 17 samples with the 286 bp and 188 bp peaks together with a 383 bp FC27 allele. These stutter peaks were excluded from further analysis. The number of *msp1 *and *msp2 *fragments detected by CE was higher than with gel electrophoresis (Kruskal-Wallis test; *msp1 P *= 0.002, *msp2 P *< 0.001) (Table 3). When estimating allele frequencies, the fragments were allocated into size bins of 3 bp. For the *msp2 *IC allelic type, 127 different fragment sizes resulted in 76 alleles. For the allelic types of *msp1 *and for the FC27 type of *msp2 *binning did not affect the number of alleles. In total 48 different *msp1 *and 96 different *msp2 *alleles were distinguished in these 223 isolates.

**Table 3:** *Msp1 *and *msp2 *genotyping by CE and gel electrophoresis of 240 microscopy *P. falciparum-*positive samples from asymptomatic children

	Capillary electrophoresis							Gel electrophoresis						
	*msp1*				*msp2*			*msp1*				*msp2*		
	K1	MAD20	RO33	total	FC27	IC	total	K1	MAD20	RO33	total	FC27	IC	total

PCR positive	195	129	124	223	169	204	223	197	129	126	223	166	201	223
Fragments^a^	314	160	124	598	230	415	645	225	147	126	498	191	253	444
Frequency^b^	33	14	1	48	20	76	96	n.d.	n.d.	n.d.	n.d.	n.d.	n.d.	n.d.
Mean^c^ (95%CI)	1.61	1.24	1.0	2.68 (2.50–2.86)	1.37	2.03	2.89 (2.66–3.13)	1.14	1.14	1.0	2.23 (2.11–2.36)	1.15	1.26	1.99 (1.89–2.09)

^a ^Total number of detected *msp1 *and *msp2 *fragments

^b ^number of different alleles detected in all samples, size bins of 3 bp

^c ^Number of alleles/sample, excluding all parasite-negative samples

n.d. not determined

### Distinguishing recrudescence from reinfection in anti-malarial drug trials

Paired blood samples (n = 114) collected on filter paper from 57 children enrolled in an anti-malarial drug trial in Zanzibar were analysed to determine recrudescence versus reinfection in follow-up samples. An initial *msp*2 typing was followed by *msp1 *typing. Successful amplification in paired samples was achieved in 53 (91.4%) and 52 (89.7%) children by CE and gel electrophoresis, respectively. Classifying outcomes based on *msp2*, resulted in 11 and 13 recrudescent cases and 41 and 38 new infections using CE and gel electrophoresis, respectively. When outcomes were reclassified by adding data from the *msp1 *genotyping, the same five infections were considered recrudescent by both techniques (Table 4).

**Table 4:** Stepwise genotyping of *msp2 *and *msp1 *in paired samples collected from children with uncomplicated *P. falciparum *malaria participating in an anti-malarial drug trial in Zanzibar.

			*msp2*		*msp1*		
Outcome	Child (study ID)	Day^a^	FC27^b^	IC^b^	K1^b^	MAD20^b^	RO33^b^
Recrudescences	K152	0	-	**599.84** 505.26	-	-	**157.51**
	K152	21	-	**599.76**	-	-	**157.50**
	K97	0	-	**559.63**	**246.15**	-	-
	K97	21	-	**559.62**	**246.05**	-	-
	M107	0	-	**523.93**	-	**220.00**	-
	M107	21	-	**523.93**	-	**220.00**	-
	M131	0	-	**487.40**	-	**202.01**	-
	M131	21	-	**487.33**	-	**202.01**	-
	M152	0	-	**528.70**	**219.25**	-	-
	M152	21	-	**528.61**	**219.26**	-	-
New infections	K108	0	**336.25**	515.38	-	-	157.53
	K108	42	**336.25**	-	246.43	-	-
	K149	0	**336.30**	452.49	182.77	201.89	-
	K149	28	**336.24**	580.85	300.79	210.91	-
	M161	0	**336.30**	531.02	138.73 146.36	210.81 220.07	-
	M161	21	**335.75** 419.85	622.87	182.72 210.17	192.88	157.62
	M164	0	**336.30** 419.71	632.64 620.97	228.45	-	-
	M164	28	**336.30**	560.93	255.70 264.91	-	157.53
	M165	0	335.91 **373.27**	464.33	210.07	210.81	157.61
	M165	28	**372.97** 419.71	-	228.28	-	-
	M173	0	**372.85**	531.85	246.31	220.0 228.15	-
	M173	42	**372.14**	499.35 564.54	201.10 264.10	246.88	-

Outcome classified by *msp2 *followed by *msp1 *genotyping to confirm recrudescence (bold) or new infection.

^a ^Paired blood samples collected before initiation of anti-malarial treatment (day 0) and at day of recurrent parasitaemia (days 21–42)

^b ^Fragment sizes in bp

- negative result

## Discussion

A widely used method for genotyping *msp1 *and *msp2 *of *P. falciparum *parasites was here adapted to multi-coloured capillary electrophoresis (CE) [7,16] resulting in improved size resolution as well as throughput compared to the original gel electrophoresis-based assay. Although substantial optimization to overcome various artifacts and practice in interpretation was required, CE clearly represents an improved technique for fragment sizing compared to sizing by gel electrophoresis.

One of the major advantages of CE is its precision in distinguishing fragment sizes to the order of single base pairs. Although gel electrophoresis is a relatively simple method to perform, size determination of PCR products is restricted by low resolution. While small differences between fragments < 10–20 bp may be distinguished by naked eye, the exact fragment size is difficult, if not impossible, to determine, especially with increasing distances to the size marker (illustrated in Figure 3). Moreover, interpreting the number of bands may vary between different readings [19]. Even with the aid of software used for size calling, variations in repeated measurements (2–16 bp) were observed in this study. To achieve a higher discrimination power, an alternative method based on RFLP has been used to analyse individual patterns of restriction fragments instead of simply sizing PCR products [20]. The advantage of PCR-RFLP compared to standard gel electrophoresis laid in the higher resolution of the generally small restriction fragments; however, a major disadvantage was the highly complex banding patterns generally found in areas of high malaria endemicity and high multiplicity of infection. In the CE assay, a size marker is added to each individual sample, which allows for high precision and reproducibility in sizing. Indeed, in this study size calling of individual alleles differed only by < 0.5–1 bp within and between separate PCR and CE runs, as well as between different instruments.

The sensitivity of detection of single laboratory lines was similar between CE and gel electrophoresis, with successful detection of 5 to 10 parasites/μl. The CE method was, however, better at detecting multiple alleles in laboratory and field samples. Similar finding was reported in a study from a different area in Ghana when another CE-based method for *msp2 *genotyping was compared to a PCR-RFLP assay [13]. Detection of these additional alleles may be explained by the higher resolution of CE, which enables distinction of the extensive diversity of the *msp1 *and *msp2 *genes that is not detected by gel electrophoresis. The high number of alleles distinguished here among Ghanian children indeed reveals an extensive polymorphism in this population. To determine allele frequencies, fragments were allocated to size bins of 3 bp since *msp1 *and *msp2 *are coding genes. When applying this strict allele definition for determining allele frequencies the 127 *msp2 *IC fragments resulted in 76 different alleles. Fragments allocated to the same size bin may still differ in sequence and represent separate alleles.

The CE method showed a high specificity in the analysis of infections with high parasite densities, whereas gel-based analyses often generated non-specific bands and smears (Figure 2), thus complicating the interpretation, especially in studies of clinical malarial infections. These larger, non-specific fragments are likely to be caused by improper re-annealing after PCR amplification reaches plateau, which may result in misaligned PCR fragments of higher molecular weight that are detected by gel electrophoresis [21]. The larger, non-specific bands are not detected under denaturated conditions during CE, as demonstrated by the detection of one single peak in monoclonal infections independent of parasite densities (Figure 2). Carry-over products from the primary PCR may also result in larger non-specific bands. However, most non-specific fragments detected on gels were too small to represent amplification products from the primary PCR of the *msp *genes.

The ability to distinguish recrudescent parasites from new infections in an anti-malarial drug trial was similar by CE and gel electrophoresis, resulting in the same outcome classification. All sizes estimated by CE had high precision and the interpretation was straightforward. Fragments of the same length may, however, still represent different clones considering that the methods only provide the length and not the sequence of the PCR products [13]. However, the probability of two alleles being classified as the same is smaller using CE. Interpretation of gels by the naked eye is more subjective and requires that paired samples from an individual, collected before treatment and during follow up, are run in adjacent lanes on the same gel. A great advantage with CE is that samples can be randomly set up in the 96-well plates, simplifying handling and decreasing the risk of cross-contamination. The reproducibility of the method also allows for the determination of allele frequencies in a study population. In the MMV/WHO guidelines for genotyping in anti-malarial drug trials, CE-based methods are recommended for fragment analysis [1]. The advantage of the method presented here is its origin in a well-established genotyping protocol [16] with the same primers both in the fluorescent and the original assay.

Quantifying the proportion of different clones within an infection is of particular interest in studies of within-host dynamics and can be used, for example, to study if a dominant clone is the cause of a clinical episode of malaria. A CE-based *msp2 *genotyping assay with single non-allele specific PCR, has previously been used to quantify the proportions of clones in individuals over time [14] Quantification by PCR is preferably determined during the exponential phase of a single round of amplification. This is supported by the poor accuracy of quantification in mixtures with multiple and different types of lines using the current nested PCR, and therefore quantification is not recommended by this method.

Setting up the CE method required extensive optimization of the original PCR protocols to overcome different types of artifacts. Although methods must always be adjusted to individual laboratories, the process reported here may be useful for other researchers who wish to adapt CE genotyping assays. In essence, the modifications involved primer concentrations and number of cycles in the nested PCR, diluting the final products in sterile water prior to CE, and setting a cut-off value of 300 rfu [13]. The characteristic companion peaks, so far observed in only a few runs, are probably of fluorescent origin and were removed by diluting the PCR products prior to CE. In completely automated assays, these artifact peaks may cause a problem since their rfu values may well be above the set cut-off. Also, the stutter peaks may coincide with true allele peaks; however, the stutters often constitute < 10% of the height of the true allele, and if high intensity peaks appear within these patterns they should be considered as true alleles. The repetitive stutter peaks are likely to represent slippage of *Taq *DNA polymerase during template extension [22], resulting in stutter fragments lacking one or several of the repetitive units. Although decreasing the number of PCR cycles in the nested reaction reduced their frequency, they were not completely eliminated through any optimization step. In view of these artifacts, we recommend that all electropherograms be initially screened manually.

When setting up the CE-based assay, the aim was to optimize throughput to reduce time and costs, as well as to simplify handling. This included (i) the use of 96-well plates in all consecutive steps from DNA extraction to CE; (ii) multiplex amplification of the outer *msp1 *and *msp2 *blocks in the primary reaction; (iii) multiplexing the CE runs with three and two colours for *msp1 *and *msp2*, respectively; and (iiii) multiplexing the nested PCR for *msp1 *and *msp2*. For *msp1*, the typing results correlated well between the simplex and multiplex nested assays. However, for the *msp2 *marker, a higher number of fragments were detected by the multiplex approach. Some of these fragments might have been genetic hybrids [23], however most were likely artifacts including *in vitro *recombination [24]. Except for the *msp2 *nested PCR, all steps of CE-based genotyping could thus be multiplexed to increase throughput. The cost of the CE based genotyping assay was similar to gel based genotyping, including reagents (PCR primers, size marker etc) and excluding equipment (e.g. DNA sequencer) and personnel.

## Conclusion

The CE-based genotyping assay described in this report allowed for precise and highly reproducible typing and sizing of *P. falciparum msp1 *and *msp2 *alleles. There is however several artifacts, inherent with fluorescence, that need to be consider in the interpretation and optimization process. The improved sensitivity and specificity of allelic discrimination makes the technique an important tool in future molecular epidemiology studies as well as anti-malarial drug trials. Additionally, more detailed and reliable determination of alleles will also contribute to the knowledge of the frequency of the different allelic types of *msp1 *and *msp2 *in different epidemiological setting

## Abbreviations

*msp*: merozoite surface protein gene; CE: capillary electrophoresis; rfu: relative fluorescent unit; PCR: polymerase chain reaction.

## Competing interests

The authors declare that they have no competing interests.

## Authors' contributions

AL planned the study, carried out the molecular work, performed the statistical analyses and wrote the paper. LW helped in optimizing the method and in the design of the study. NF participated in the study design. MK provided the patient samples from Ghana. AM provided the patient samples from Zanzibar, Tanzania and reviewed the manuscript. IF participated in the design of the study and reviewed the manuscript. AF planned the study, interpreted the results and wrote the paper. All authors read and approved the final manuscript.

## Supplementary Material

Additional file 1 **Relative quantification of *msp2 *IC allelic type alleles by capillary electrophoresis in mixtures of different proportions of laboratory lines**. This file contains the relative quantification of mixtures of laboratory lines of the *msp2 *IC allelic type in different proportions. Click here for file

Additional file 2 **Relative quantification of *msp2 *FC27 and IC allelic type alleles, i.e. labeled with different colors, by capillary electrophoresis in mixtures of different proportions of laboratory lines**. This file contains the relative quantification of mixtures of laboratory lines of the *msp2 *IC and FC27 allelic types in different proportions. Click here for file
