# Supplementary material for: Optimization and validation of multi-coloured capillary electrophoresis for genotyping of Plasmodium falciparum merozoite surface proteins (msp1 and 2)
Source: Malar J. 2009 Apr 23;8:78. doi: 10.1186/1475-2875-8-78 (PMC2680902; doi:10.1186/1475-2875-8-78)
Supplement: Additional file 1 — Relative quantification of msp2 IC allelic type alleles by capillary electrophoresis in mixtures of different proportions of laboratory lines. This file contains the relative quantification of mixtures of laboratory lines of the msp2 IC allelic type in different proportions. [file 1475-2875-8-78-S1.doc]

**Additional file 1**

**Table S1.** Relative quantification of *msp2* IC allelic type alleles by capillary electrophoresis in mixtures of different proportions of laboratory lines

| Lines | Parasites/µl | Ratio  in mixtures | Calculated ratio  (CE peak heights) | Calculated ratio  (CE peak AUC) |
| --- | --- | --- | --- | --- |
| F32/TM180 | 5000/50 | 99:1 | 89: 11 | 89: 11 |
| F32/TM180 | 5000/50 | 99:1 | 86: 14 | 85: 15 |
| F32/TM180 | 5000/50 | 99:1 | 83: 17 | 83: 17 |
| Average |  |  | 86:14 | 86:14 |
|  |  |  |  |  |
| F32/TM180 | 500/500 | 50:50 | 42: 58 | 42: 58 |
| F32/TM180 | 500/500 | 50:50 | 42: 58 | 42: 58 |
| F32/TM180 | 500/500 | 50:50 | 43: 57 | 41: 59 |
| Average |  |  | 42:58 | 42:58 |
|  |  |  |  |  |
| F32/TM180 | 50/5000 | 1:99 | 2: 98 | 2: 98 |
| F32/TM180 | 50/5000 | 1:99 | 2: 98 | 2: 98 |
| F32/TM180 | 50/5000 | 1:99 | 4: 96 | 4: 96 |
| Average |  |  | 3:97 | 3:97 |
|  |  |  |  |  |
| F32/TM180/7G8 | 500/500/500 | 33:33:33 | 22:75:3 | 21:76:3 |
| F32/TM180/7G8 | 500/500/500 | 33:33:33 | 21:76:3 | 21:76:3 |
| F32/TM180/7G8 | 500/500/500 | 33:33:33 | 22:75:3 | 21:76:3 |
| Average |  |  | 21:76:3 | 21:76:3 |
|  |  |  |  |  |
|  |  |  |  |  |

Results from quantifications in triplicates

AUC; area under the curve
