# Supplementary material for: Optimization and validation of multi-coloured capillary electrophoresis for genotyping of Plasmodium falciparum merozoite surface proteins (msp1 and 2)
Source: Malar J. 2009 Apr 23;8:78. doi: 10.1186/1475-2875-8-78 (PMC2680902; doi:10.1186/1475-2875-8-78)
Supplement: Additional file 2 — Relative quantification of msp2 FC27 and IC allelic type alleles, i.e. labeled with different colors, by capillary electrophoresis in mixtures of different proportions of laboratory lines. This file contains the relative quantification of mixtures of laboratory lines of the msp2 IC and FC27 allelic types in different proportions. [file 1475-2875-8-78-S2.doc]

**Additional file 2**

**Table S2.** Relative quantification of *msp2* FC27 and IC allelic type alleles, i.e. labeled with different colors, by capillary electrophoresis in mixtures of different proportions of laboratory lines

| Lines | Parasites/µl | Ratio  in mixtures | Calculated ratio  (CE peak heights) | Calculated ratio  (CE peak AUC) |
| --- | --- | --- | --- | --- |
| K1/F32 | 50000/500 | 99:1 | 59:41 | 49:51 |
| K1/F32 | 50000/500 | 99:1 | 66:34 | 56:44 |
| Average |  |  | 63:37 | 53:47 |
| K1/7G8 | 5000/50 | 99:1 | 73:27 | 65:35 |
| K1/7G8 | 5000/50 | 99:1 | 92:8 | 88:12 |
| Average |  |  | 83:17 | 77:23 |
|  |  |  |  | . |
| K1/F32 | 5000/5000 | 50:50 | 80:20 | 74:26 |
| K1/F32 | 5000/5000 | 50:50 | 89:12 | 83:17 |
| Average |  |  | 85:15 | 79:21 |
| K1/7G8 | 5000/5000 | 50:50 | 44:56 | 35:64 |
| K1/7G8 | 5000/5000 | 50:50 | 41:59 | 30:70 |
| Average |  |  | 43:57 | 33:67 |
|  |  |  |  |  |
| K1/F32 | 500/50000 | 1:99 | 65:35 | 56:44 |
| K1/F32 | 500/50000 | 1:99 | 60:40 | 49:51 |
| Average |  |  | 63:37 | 53:47 |
| K1/7G8 | 50/5000 | 1:99 | 78:22 | 71:28 |
| K1/7G8 | 50/5000 | 1:99 | 32:68 | 23:77 |
| Average |  |  | 55:45 | 47:53 |
|  |  |  |  |  |
|  |  |  |  |  |

Results from quantifications in duplicates

AUC; area under the curve
